# Short-term effects of postural taping on pain and forward head posture: a randomized controlled trial

**DOI:** 10.1186/s12891-022-05083-5

**Published:** 2022-02-19

**Authors:** Sofia Ryman Augustsson, Sara Reinodt, Evelina Sunesson, Emma Haglund

**Affiliations:** 1 Department of Sport Science, Faculty of Social Sciences, Linnaeus University, SE-391 82 Kalmar, Sweden; 2 Halmstad University, School of Health and Welfare, Halmstad, Sweden; 3 Spenshult Research and Development Centre, Bäckagårdsvägen 47, SE-302 74 Halmstad, Sweden; 4 Rydberg Laboratory of Applied Sciences, Halmstad University, Halmstad, Sweden; 5 Department of Clinical Sciences, Section of Rheumatology, Lund University, Lund, Sweden

**Keywords:** Balance body tape, Posture analysis, Neck pain, Ergonomics, Movement behavior

## Abstract

**Background:**

Balance Body Tape (BBT) is a recently developed taping-method with the aim to reduce pain and improve posture through change in movement behavior. However, the potential effects of a treatment with BBT are scarcely documented. Therefore, the aim with this study was to investigate the effect of a three-week Balance body tape-treatment on the intensity of perceived neck, shoulder and back pain and forward head posture.

**Methods:**

In this RCT study, subjects (*n* = 26), who reported being university students or having a sedentary work and experiencing pain in neck, back or shoulders, were randomized to either an intervention (*n* = 12) or control group (*n* = 14). The intervention group received a three-week treatment with BBT, the control group received no treatment. A questionnaire regarding pain, including a Numeric Rating Scale (NRS) measuring pain intensity, and a Photographic posture analysis measurement (PPAM) regarding the craniovertebral (CV) angle were assessed before and after the intervention for both groups. Wilcoxon’s signed rank test and Mann-Whitney U test was used to assess intra- and between group differences respectively. The relationship between pain intensity and CV angle was assessed using Spearman’s correlation.

**Results:**

No difference in demographic and physical characteristics between the groups were noted at baseline (*p* > 0.05). Pain intensity at baseline was 5 for the intervention group and 4 for the control group (*p* = 0.330). At follow up, the intervention group reported a lower score (NRS = 2.5, *p* = 0.003) whereas the control group had no significant difference in pain intensity (NRS = 3, *p* = 0.086). No significant change was found in the CV angle (*p* = 0.058) and no correlation was found between NRS and the CV angle (*r* = 0.102, *p* = 0.619).

**Conclusion:**

A short treatment period with BBT may, compared to no treatment, have a small reducing effect on pain intensity in neck, back and/or shoulders. However, no effect was found on forward head posture in this study.

**Trial registration:**

Registered retrospectively on 08/11/2021. NCT05111704.

Trial registration page link:

## Background

A neutral, or ‘good’ posture can be defined as a state of musculoskeletal balance [1]. In this state, the different parts of the body are arranged in relation to each other so that a minimal effort is required to maintain this position and the muscles can work efficiently. Prolonged sitting, especially during non-ergonomic conditions, seem to be a risk factor for poor posture and pain [2]. For example, spending more than 5 h a day in front of a computer have been shown to be associated with severe neck pain [3]. A poor posture can cause myofascial pain and change in movement behavior due to increased stress on posterior structures or by a prolonged static loading of muscles, tendons and joints [3].

One type of poor posture that is associated with neck pain is the forward head posture [3–5]. The common definition of forward head posture is that the head is, in relation to the vertical line of the body’s centre of gravity, held in an anterior position [3]. This can be assessed from measuring the craniovertebral (CV) angle from a photograph taken in the sagittal plane. A horizontal line through the seventh cervical vertebra (C7) and one line from the tragus of the ear to C7 form together the CV angle [6]. The CV angle in healthy subjects is approximately 48–50 degrees [4, 7]. A smaller CV angle indicates a more forwarded head posture. A high intra-rater and inter-rater reliability has been noted when using this method for measuring the CV angle (ICC between 0.81 to 0.87) [4].

Lau et al. [4] showed that subjects with neck pain more often have a smaller CV angle (mean 40.13 ± 6.68 degrees) than subjects without neck pain (mean 48.40 ± 5.52 degrees), but also that the CV angle was negatively correlated with pain intensity in the group with neck pain (*r* = − 0.36 *p* = 0.06). A more recent study found a moderate negative correlation between CV angle and pain intensity (*r* = − 0.536, *p* < 0.01) and weak negative correlation (*r* = − 0.389, *p* < 0.01) between CV angle and disability due to neck pain [3]. The authors are proposing that a smaller CV angle can be related to greater pain intensity and disability and conclude that improving head posture is of high importance in patients with neck pain. Patients with chronic neck pain have also been demonstrated to have a different movement behavior compared to healthy controls [8].

There are several treatments with the aim to improve posture and posture-related pain. For example, ergonomic modifications in an office environment, motor control training, body awareness training and strength-training [9]. Kinesiology Tape is another potential treatment-method used to reduce pain and neck disability and improve posture and movement behavior [10, 11]. Kinesiology tape or Kinesio Taping (KT) can provide support and stability to muscles and joints without limiting range of motion due to its elasticity [12]. KT may also have a small beneficial role in improving strength and range of motion in injured athletes [13]. KT have been shown to give a greater reduction in neck disability index (NDI) than postural correction exercises, when short-term effects were studied [14].

The mechanisms behind how KT may improve posture and reduce pain are not completely established [10, 15]. Improvement of circulation and providing a positional stimulus to the skin, muscle or fascia as well as providing afferent input to the central nervous system are examples of mechanisms that have, by the developer of KT, been suggested to be contributing factors for change in movement behavior [11, 13].

Balance Body Tape (BBT) is a taping method developed by the company Babota AB [16]. BBT is, according to the company, a tighter and less elastic tape compared to KT [17]. The “unique” elasticity of BBT is supposed to make sure that it is muscle activation, and not a passive treatment, that ensure a better posture [16, 17]. Except for reacting to touch, pressure or stretch in the skin, the mechanoreceptors are also sensitive to stimulation of muscles and joints which means that they help keeping track of the movement of the body [18]. The BBT is available as different pre-cut pieces depending on treatment purpose, and as tape rolls. The aim with the pre-cut posture-BBT is having a positive impact on upper body-posture and the lower back-BBT is supposed to help keeping a good pelvic tilt. The aim with the BBT treatment is a change in movement behavior and postural improvements, that may result in benefits such as less pain or discomfort from the back, neck and shoulders [16].

Taken together, prolonged sitting and poor postures seems to be related to pain due to increased stress on muscles, tendons and joints [3, 4, 19]. Previous studies are proposing that a smaller CV angle can be related to greater pain intensity and that improving posture may be an important part of the treatment of pain in neck, back or shoulders [3, 4]. BBT is a recently developed taping-method with the aim to improve posture and reduce pain through a change in movement behavior [16]. To our knowledge, no previous study has investigated or documented the potential effects of a treatment with BBT.

Thus, the aim with this study was to investigate the effect of a three-week Balance body tape-treatment on the intensity of perceived neck, shoulder and back pain and forward head posture.

### Study hypotheses

Primary hypothesis: There would be significant improvements in pain after three-week of Balance body tape-treatment in the intervention group and changes would be more prominent compared to the control group.

Secondary hypothesis: There would be significant improvements in forward head posture with a larger CV angle in the intervention group compared to the control group.

## Materials and methods

### Study design

Data for this randomized controlled trial (RCT) study, adhering to the CONSORT statement [20, 21], included a short treatment period (19 days) of postural taping with Balance Body Tape (BBT). Subjects were randomly assigned, by lottery, into intervention- and control group. The subjects in the intervention group received treatment with BBT whereas the subjects in the control group received no treatment. A questionnaire regarding pain and a Photographic posture analysis measurement (PPAM) of habitual sitting posture, focusing on neck posture, were accomplished at baseline and after 22 days for both groups.

### Subjects

**Fig. 1:**
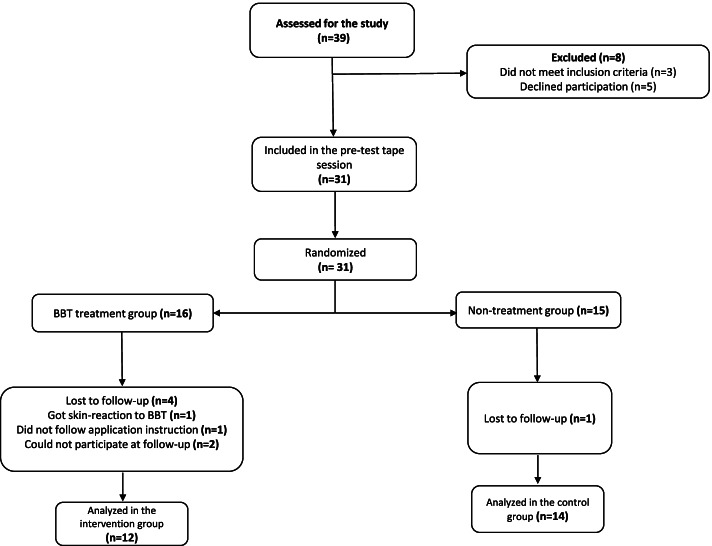
Flow chart of inclusion process of subjects for treatment with Balance Body Tape (BBT) and for the control group

Subjects were recruited by advertisements at a University in the southern part of Sweden, through the Student Union and a Business Incubator on the University area and on social media (Facebook and Instagram). Subjects that showed interest to participate (*n* = 39) received more information about the study through e-mail or a printed information paper. The study included subjects aged 18–39 who reported being a university student or having a sedentary work and at inclusion had at least a 2-point score at the Numeric Rating Scale [22] regarding to self- perceived pain, ache, discomfort or fatigue in neck, shoulders or back associated with prolonged sitting [23]. Subjects who had suffered from severe back or neck injuries within the last 3 months or had an ongoing treatment plan for any back or neck problems were excluded. Subjects that had severe contact allergy or eczema or got skin reaction at the first application and, subjects who had previously tried BBT, were also excluded. Thirty-one subjects where finally included in the study and were randomly assigned into intervention- (*n* = 16) or control group (*n* = 15) (Fig. 1). The procedures as well as any possible risks of participating were explained to all subjects before the start of the study both oral and by written information. Written consent for participation was obtained in accordance with research ethics guidelines.

### Demographic and physical characteristics assessments

To describe demographic and physical characteristics of subjects, self-reported level of physical activity and daily time sedentary, Body Pain Diagram and The Neck Disability Index was assessed at baseline for all subjects.

#### Physical activity

To assess self-reported level of physical exercise, physical activity (per week), and sedentary time (per day), indicator issues from The National Board of Health and Welfare were used [24]. The level of physical exercise was measured in minutes per week on a six-graded scale; 0 min, less than 30 min, 30–60 min, 60–90 min, 90–120 min, and more than 120 min. Physical exercise refers to activity that contributes to shortness of breath, such as running, gymnastics or ball sports. Level of physical activity was measured in minutes per week on a seven-graded scale; 0 min, less than 30 min, 30–60 min, 60–90 min, 90–150 min, 150–300 min, and more than 300 min. Physical activity refers to low intensity activities such as walking, cycling or gardening. Sedentary time was measured in hours per day on seven-graded scale; never, 1–3 h, 4–6 h, 7–9 h, 10–12 h, 13–15 h, almost all day. Sedentary time refers to the time spent on resting/sitting during a normal day (apart from sleep).

#### Body pain diagram

A Body Pain Diagram (BPD) [25] was used to examine the location of pain. On the BPD, the body was divided into 18 different areas. For each area, the subjects were asked to score how often they perceived pain on a 6-point scale where 0 = never and 6 = almost every day.

#### Neck disability index

The Neck Disability Index (NDI) [26] was used to indicate to which extent the pain affects subjects in their daily life. The NDI consists of ten sections with five statements scored from 0 to 5 (0 = no pain, 5 = worst pain imaginable). A total score on the NDI is calculated by adding up the points from all ten sections (maximum 50 points) [26]. From the total amount of points, score disability level is rated as follows: 0–4 = No disability; 5–14 = Mild disability; 15–24 = Moderate disability; 25–34 = Severe disability; 35–50 = Completely disabled.

### Testing procedure and randomization

First subjects’ age, gender, height and weight were collected. Next, the subjects filled out a questionnaire including both demographic information as well as a Numeric Rating Scale (NRS) [22], Body Pain Diagram (BPD) [25] and Neck Disability Index (NDI) [26]. The test managers were available if any uncertainties about the questionnaire occurred. When the questionnaire was completed a six-minute computer work session, including photographing of the habitual sitting posture in the sagittal plane, was performed. Both measurement occasions followed the same test protocol and was performed in a secluded room. The subjects were informed if they had been randomized into intervention- or control group when the test protocol was completed at the first measurement occasion. Simple randomization was used for group allocation where each subject chose a sealed note in which one of the words “intervention” or “control” was written. One of the researcher carried the randomization. The intervention group got instructions about the BBT treatment schedule and the application procedure. The first set of BBT was applied by the test managers on subjects’ shoulder and lower back before a mobile photograph was taken and sent to the subject to have as a reference for upcoming applications at home. Both groups were informed to not take any kind of treatment such as massage or chiropractic during the study.

### Outcome measures

#### Numeric rating scale

The primary outcome measure was pain which was assessed by the Numeric rating scale (NRS) [23]. The subjects were asked to assess their intensity of pain in neck, shoulders or back during the last week. To be considered a clinically relevant change in pain intensity level between baseline and follow-up measurement, at least a two-point change on the NRS was needed. This has been shown to be the minimal clinically important difference (MCID) [23].

#### Photographic posture analysis method

**Fig. 2:**
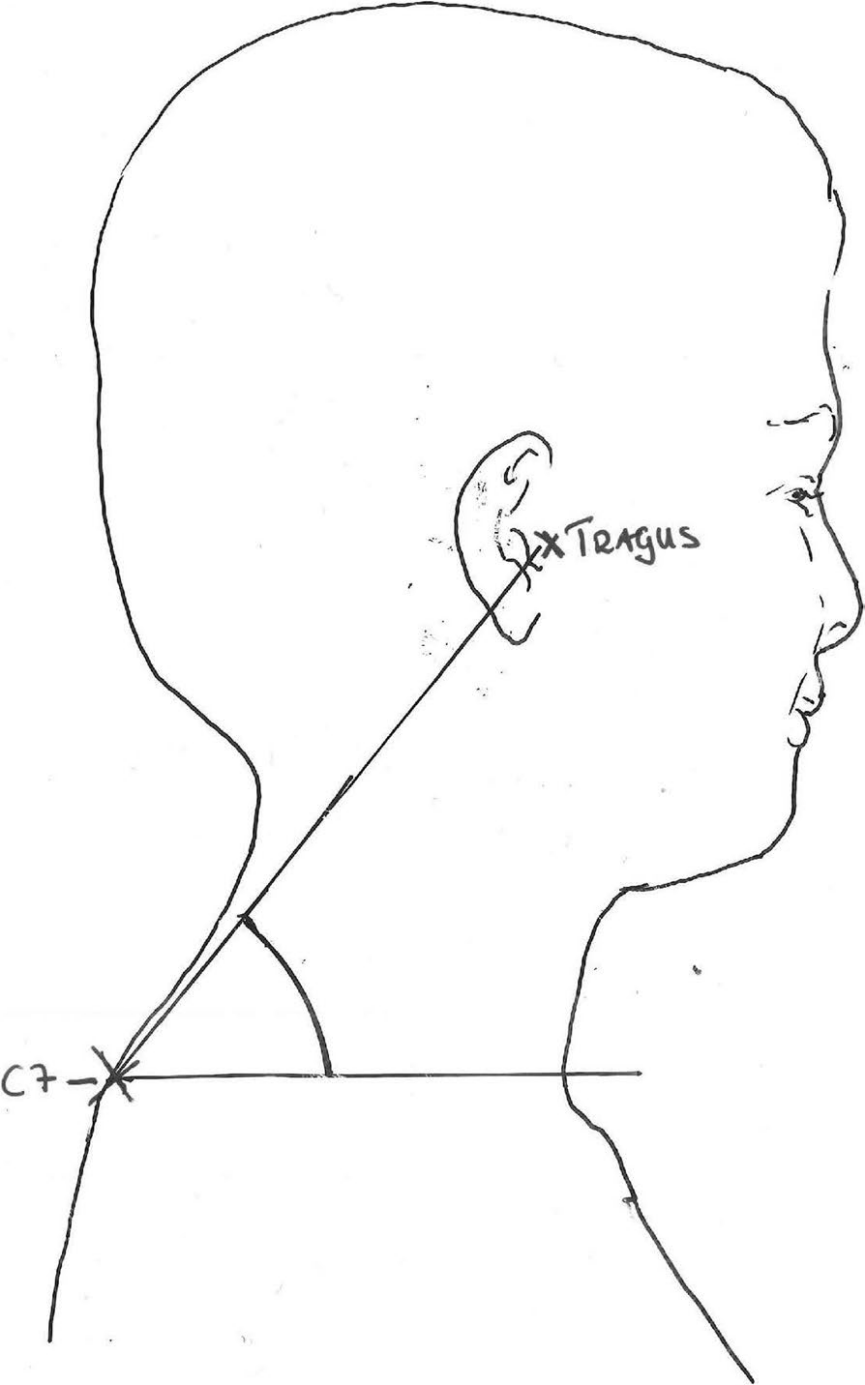
Anatomical landmarks at C7 and tragus forming the craniovertebral angle

Secondary outcome measure was forward head posture. Using the Photographic Posture Analysis Method (PPAM) to evaluate sitting posture is considered a valid and reliable method [27]. The craniovertebral (CV) angle was assessed from photographs of the habitual sitting posture taken in the sagittal plane at starting position and then every 2 min during a 6-min computer task in a simulated computer workstation [19, 27]. For each subject, preparations consisted of positioning anatomical markers, using coach tape, on the spinous process of the seventh cervical vertebra (C7). The starting position was standardized following a protocol described by Falla et al. [19]. Subjects were placed in an upright position defined as “a vertical pelvic position (no anterior or posterior pelvic tilt) and with the assumption of a lumbar lordosis and thoracic kyphosis” [19]. Subjects had their feet flat on the floor and the knees in 90 degrees of flexion with arms hanging relaxed by the side. During the 6-min computer task, subjects were instructed to play the game Klondike Solitaire, using their dominant hand and have the other hand rested on the desk. They were instructed to work in their natural work-position [3]. The subject was instructed not to cross the thighs or move the chair until the test manager interrupted the session. The simulated workstation consisted of a 72 cm high table, where a 14″ laptop was placed 11 cm from the table edge. A non-adjustable chair (height 45 cm, seat depth 40 cm) was used. The front edge of the chair was placed in line with the table edge. A high definition mobile camera (Huawei P10 Leica Summarit-H 1:2.2/27 ASPH, Huawei Technologies Co., Longgang District, Shenzhen, China) was mounted 90 cm from the floor on tripods placed 1,2 m away from the subjects’ chair and all anatomical markers were visible in one image. The Android application ‘Silent Camera’ and a Bluetooth remote control (Holdit Remote Shutter) were used while taking the photographs so that the test manager could sit further away from the subject than 1.2 m. The digital clock on the computer, and in the game, were covered with white sport tape so as not to distract the subject. The same equipment was used for all measurements and tape-markers on the floor made sure that the equipment was placed in the same way during all measurements. All photographs were taken from the subjects’ right side. The postural analysis was performed using the free software Kinovea (version 0.8.15, Creative Commons Attribution 3.0). On each picture (taken minute 0, 2, 4, 6), 190% digital zoom was applicated. A digital marker was positioned on the anatomical markers on vertebrae C7 and on the tragus of the ear, before a horizontal line were drawn through the C7-marker (Fig. 2) [6]. Another line was drawn through C7 and tragus. The CV angle was measured in degrees ( °) between the two lines. The same measurement procedure was done with each picture (minute 0, 2, 4, 6). CV angle during minute 0–6 were measured and expressed in absolute degrees and a mean value of the CV angle during minute 2–6 was calculated.

### Intervention

#### Balance body tape-treatment

BBT is waterproof, latex free, made of 100% cotton and is attached to the body with non- toxic glue [16]. The study included posture taping during a 19-day treatment period with posture- and lower back BBT. The lower back taping was performed on the grounds that it would capture an ideal alignment in the sagittal plane [1]. Follow up- measurement was accomplished 22 days from baseline measurement and treatment initiation. The treatment protocol was 72 h with BBT applicated on shoulders and lower back which were followed by 24 h of rest before a new set of BBT were applicated for another 72 h [16]. This procedure was repeated with five sets of BBT.

The test managers accomplished the first application of BBT, during the first measurement occasion, after the subjects’ baseline measurements were completed. The four remaining applications were completed at home, following an individual schedule and with assistant help. Subjects were instructed to perform the application about the same time (+/− 2 h) each time and to make sure that all four applications were carried out at the day and time instructed. Application instructions, both verbal and written, were provided at the first measurement occasion and the test managers were available to answer questions on email and phone during the whole intervention.

Application procedure: The application started by cleansing the skin and, if necessary, removing hair growth in order for the tape to sit well. The protective paper was removed and both tops were applied just below the neck hair. Then, one side of the protective paper was removed and the tape was attached in the order; neck, shoulders and thoracic spine. For the lower back the tape was attached from top to bottom. To ensure that the glue adheres properly, the tape was rubbed by hand. The head was kept in a neutral position throughout the application procedure.

### Statistical analyses

The analysis was performed using Microsoft Excel and IBM SPSS (IBM SPSS Statistics for Windows, Version 24.0. IBM, Armonk, New York, USA). The level of significance was determined to *p* < 0.05. Due to the relatively small sample size and the qualitative nature of the NRS (ordinal data), non-parametric tests were used. The Spearman’s rank-order correlation was used to examine the correlation between score on NRS and the CV angle. The correlation coefficient (r^s^) were categorized as follows: 0.00–0.19 Very weak, 0.20–0.39 Weak, 0.40–0.59 Moderate, 0.60–0.79 Strong and 0.80–1.00 Very strong [3]. Wilcoxon’s signed rank test was used to assess differences in NRS and CV angle before and after the treatment period, within the groups. Mann-Whitney U test was used to assess differences between intervention- and control group before and after the intervention. The change in score before and after the intervention was also compared to the minimal clinically important difference (MCID = 2) [23]. The number of subjects was established on sample size calculation with the NRS as the primary outcome measure. Based on a power of 0.90 (α =0.05), approximately 13 subjects would be required, in each group (intervention and control, respectively), to detect a minimal clinical relevant difference change in pain intensity level of at least a two-point change on the NRS between groups. Therefore, this study was planned to recruit a minimum of 30 subjects with regard for potential dropouts. Allowing a drop-out rate of about 15%, 16 subjects in the intervention group and 15 in the control group were recruited.

## Results

### Demographic and physical characteristics

A total of 26 subjects fulfilled the study, 20 women and six men with a mean age of 25 years (21–36). The baseline demographic and physical characteristics of the intervention- and control group shown in Table 1 were similar between the groups (*p* > 0.05). For the whole group (*n* = 26) 20 subjects reported being students and six having a sedentary work. Median score on the Neck Disability Index was 9.5 for the whole group, which indicates a mild disability. The median pain intensity at baseline was 4.0 at the Numeric Rating Scale.

**Table 1:** Demographic and physical characteristics at baseline measurement (n = 26)

	All subjects (***n*** = 26)	Intervention group (***n*** = 12)	Control group (***n*** = 14)
	Mean (SD)	Mean (SD)	Mean (SD)
**Age (years)**	25 ± 3.5	25.8 ± 3.0	24 ± 3.8
**Height (cm)**	172 ± 3.5	172.2 ± 4.9	172.2 ± 8.1
**Weight (kg)**	72 ± 12.8	73.4 ± 11.0	71.6 ± 14.1
**Craniovertebral angle**	35.5° ± 9.5	33° ± 11.2	37° ± 7.8
**Female/male (n)**	6/20	9/3	11/3
	Median (min-max)	Median (min-max)	Median (min-max)
Level of physical exercise (0–5)*	4 (0–5)	4 (1–5)	3.5 (0–5)
Level of physical activity (0–6)**	4 (2–6)	5 (2–6)	3.5 (2–6)
Level of time sedentary (0–6)***	3 (1–6)	3 (1–6)	3.0 (2–6)
**NDI (0–50, no disability to complete)**	9.5 (0–19)	7 (2–14)	10 (0–19)
**NRS (0–10, best to worse)**	4 (2–7)	5 (3–7)	4 (2–7)

*0 = 0 min to 5= > 120 min/week **0 = 0 min to 6= > 300 min/week, ***0 = “Never”, 1 = 1–3 h/day, 2 = 4–6 h/day, 3 = 7–9 h/day, 4 = 10–12 h/day, 5 = 13–15 h/day, 6 = Almost all day”

*NDI* Neck Disability Index, *NRS* Numeric Rating Scale

The BPD showed that the subjects reported perceived neck pain more than once a week (4.0), pain in upper back once a week (3.0) and lower back pain once a week (3.0) with no significant differences between intervention and control group (*p* > 0.05) at baseline (Table 2).

**Table 2:** Median frequency of self-reported pain in neck, upper back and lower back at baseline assessed from Body Pain Diagram (*n* = 26)

	All subjects (***n*** = 26)	Intervention group (***n*** = 12)	Control group (***n*** = 14)
**Frequency of pain**	Median (min-max)	Median (min-max)	Median (min-max)
**Neck (0–5)**^a^	4 (0–5)	4 (2–5)	3.5 (0–5)
**Upper back (0–5)**^a^	3 (0–5)	3 (0–5)	2.5 (0–5)
**Lower back (0–5)**^a^	3 (0–5)	3 (0–5)	3 (1–5)

^a^0 = never, 1 = rarely, 2 = once a month, 3 = once a week, 4 = more than once a week, 5 = almost every day

### Effect of balance body tape-treatment on pain intensity

The intervention group had a significant lower score on NRS after the short treatment period with BBT, compared to before the treatment, from a median of 5 to 2.5 (*p* = 0.003) (Table 3). The control group had no significant difference between baseline- and follow up, from a median of 4 to 3 (*p* = 0.086). There was no significant difference between the groups NRS score, either at baseline or at follow up (*p* ≥ 0.143). However, the intervention group had a significant greater change in pain intensity than the control group (median value − 2 and − 1 respectively) (*p* = 0.008) (Table 3).

**Table 3:** Pain intensity measured with the Numeric Rating Scale (NRS) before and after intervention in intervention and control group

	Intervention group (***n*** = 12)	Control group (***n*** = 14)
**NRS**	Median (min-max)	Median (min-max)
**Baseline**	5 (3–7)	4 (2–7)
**Follow-up**	2.5 (0–7)*	3 (2–8)
**Baseline vs Follow-up**	−2 (− 7-0)‡	−1 (− 4–3)‡

NRS 0–10, best to worse

* Intragroup difference (*p* < 0.05)

‡ Difference between groups (*p* < 0.05)

### Posture analysis

**Fig. 3:**
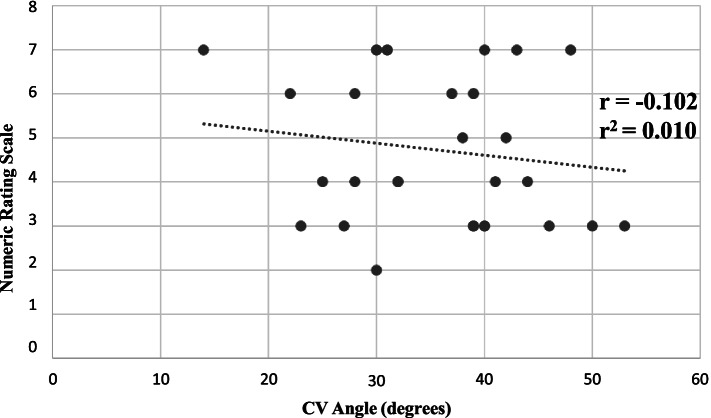
Scatterplot and trendline between score on Numeric Rating Scale (0–10, best to worse) and Craniovertebral angle (degrees) at baseline measurement (*n* = 26). Correlation coefficient *r* = − 0.102, degree of determination r^2^ = 0.010 (*p* > 0.05)

There were no differences in the CV angle, in the upright sitting posture analysis between the two groups at baseline (intervention group; mean 33° ±11.16, control group; mean 37° ± 7.8) or at follow-up (intervention group; mean 37° ± 9.4, control group; mean 37° ± 6.9) (*p* ≥ 0.110). The improvement in the CV angle, by 4°, in the intervention group, was non-significant (*p* = 0.058). No difference was found between base-line and follow-up for the control group (*p* = 0.937). The pain intensity (NRS score) at baseline had no correlation to the mean CV angle during the six-minute computer work session (*r* = − 0.102, r^2^ = 0.010, *p* > 0.05) (Fig. 3).

## Discussion

The main result of this study was that BBT treatment seems to have had a small, significant reduction on pain intensity immediately after the intervention. In addition, there was a significant difference in the change of pain intensity between the intervention- and control group. For the control group, no significant difference in pain intensity between baseline and follow up could be seen. No differences in CV angle between groups before or after the intervention and no correlation between pain intensity and CV angle was noted.

The change in pain intensity, from baseline to follow up, was, for the intervention group, equal to the MCID (2) and, thus, clinically meaningful [23]. The difference in the change of pain intensity between intervention- and control group was statistically significant, though it was not clinically meaningful. However, a one unit (− 1) difference has been defined as “slightly better” reflecting the minimum and lowest degree of improvement that could be detected [28]. Still, a NRS change score of − 2 was reported to be best associated with the concept of “much better” improvement and suggested as a clinically important outcome [28]. When comparing the intervention group results, the reduction (− 2), in pain intensity after treatment with BBT, is slightly greater than the pain reduction that have been presented in previous studies including treatment with kinesiology tape (KT) [10, 15, 29]. It is worth to note that these studies presented the results of pain intensity in mean values instead of median and that the present study had a longer treatment-period. A study that examined the pain reducing effect of pre-cut kinesiology tape in combination with exercise in subjects with subacromial impingement reported a mean change of − 0.84 on NRS at rest and − 1.46 during arm elevation after 2 weeks of treatment [10]. In subjects with neck pain due to acute whiplash injury, KT reduced pain with − 1.1 on NRS after 24 h of treatment [15]. However, the intervention (3 weeks) in the present study was longer than the treatment period in the KT studies mentioned above, which might influence the results. When 48 h of treatment with KT was compared to 48 h of placebo-treatment and no treatment in three groups of subjects with lower back pain, there was no significant or clinically meaningful differences between the groups pain intensity after either 48 h or 7 days [29]. Mauricio et al. [29] concluded that the effects of KT- treatment are” similar to the placebo effect”. The present study did not include any placebo-treatment group and can therefore not evaluate whether the reported pain reduction in the intervention group after the treatment with BBT was influenced by the placebo effect.

There was no significant improvement in the CV angle for the intervention group and no difference between groups, in the upright sitting posture analysis, during 6-min computer work. In addition, no correlation between pain intensity and CV angle was found in this study. Previous work by Lau et al. [4] reported a weak negative correlation (*r* = − 0.360, *p* = 0.06) and Subbarayalu and Ameer [3] found a moderate negative correlation (*r* = − 0.536, *p* < 0.01). The median CV angle for subjects in the present study (37.5 degrees) was smaller than what have been reported in previous research, where subjects with neck pain had a mean CV angle of 40.13 ± 6.68 degrees in one study and 41 degrees for females and 42 for males in another [3, 4]. It has previously been reported that subjects without neck pain have a mean angle of 48.40 ± 5.52 degrees, while the angle in subjects with neck pain was 40.13 ± 6.68 degrees [4]. Van Niekerk et al. [27] reported an angle of 47.66 ± 9.55 degrees when pain free subjects were instructed to sit in a normal position and 21.49 ± 27.57 degrees in slouched position. Compared to these results, the median CV angle in this study, measured from habitual sitting posture during a six-minute computer task, is smaller than previously reported mean-values measured from a normal position, but greater than the one measured in slouched position. The lack of improvement in CV angle in the present study might be due to excessively poor posture. The BBT might not be effective enough for such small CV angles as presented in the present study. Another explanation might be the short treatment period (3 week) used in the present study. However, short treatment periods have also been used in previous KT studies [14, 15]. To ensure ideal alignment in the sagittal plane [1], we choose additional application at the lower back. The cervical, thoracic and lumbar spine are all biomechanically connected to each other and for example, postural changes in either the thoracic or lumbar spine can lead to changes of the cervical lordosis [4]. A deviation from the ideal alignment and a musculoskeletal unbalance characterizes a poor posture [1]. In the two studies investigating the relationship between CV angle and pain, the mean pain intensity was reported being 5.85 on NRS and 4.89 on the VAS-scale respectively [3, 4], which may not be comparable with a median NRS-score of 4.0 in this study. Yet, it can be concluded that the correlation between smaller CV angle and greater pain intensity that have been proposed in previous research does not seem to exist in the subjects of the present study. As stated above, there was improvement in pain intensity but no improvement in CV angle. Thus, one possible explanation for the lack of correlation in the present study could be that the BBT had effect on pain but not on posture. The effect on pain might therefore be due to other factors such as the decreased pressure on subcutaneous nociceptor as a result of the tape lifting the skin [13].

One strength of the present study is the use of valid, reliable and clinically applicable methods for evaluation of treatment [22, 27]. Yet, some limitations in our study need to be acknowledged. First, the time for the intervention was only 3 weeks instead of the 6 weeks recommended by Babota AB [16]. Also, the follow-up measurement was accomplished only 3 days after the treatment was finished. This means that this study can only interpret short time effects. Further, the first set of BBT was applicated by the test managers but the following applications were performed at home which might be an issue of concern when it comes to monitoring the intervention. However, we believe that with a home based intervention the compliance increase. In addition, the idea with posture taping is that it could be used a self-administrated treatment which facilitate usage. Lastly, to evaluate the effect of BBT-treatment a comparison to placebo-treatment and/or other traditional treatments, such as motor control and strength-training protocol, may give additional valuable information.

## Conclusion

A short treatment period with Balance Body Tape may, compared to no treatment, have a small reducing effect on pain intensity in neck, back and/or shoulders. However, Balance Body Tape do not seem to have impact on forward head posture. To optimize treatment for subjects with neck pain, a longer therapy period with Balance Body Tape might be needed. In addition, the effect over time needs to be addressed in future studies.

## Data Availability

The data collected and analyzed in the current study are not publicly available due to ethical restrictions, but are available from the corresponding author upon reasonable request.

## Abbreviations

BBT: Balance Body Tape
NRS: Numeric Rating Scale
PPAM: Photographic posture analysis measurement
CV: Craniovertebral
C7: Seventh cervical vertebra
BPD: Body Pain Diagram
NDI: Neck Disability Index
KT: Kinesiology tape
ICC: Intraclass correlation coefficient
MCID: Minimal clinically important difference
